# Buffalo Dental Association

**Published:** 1865

**Authors:** Geo. B. Snow


### BUFFALO DENTAL ASSOCIATION.

The first annual meeting of the Buffalo Dental Association was held at their rooms on Monday Evening, June 5th. After the usual preliminary business was transacted, the members present proceeded to ballot for officers for the ensuing year. The election resulted as follows:

DR. R. G. SNOW, *President.*
" B. S. BROWN, *Vice-President.*
" GEO. B. SNOW, *Secretary.*
" A. R. SOUTHWICK, *Treasurer.*

The retiring President, Dr. Geo. E. Hayes, then read an address, a copy of which was requested for publication.

After the induction of officers, the following subject was taken up for discussion: "The best method of taking impressions." After a very interesting discussion, the meeting adjourned to meet on the first Monday evening in July.

GEO. B. SNOW, *Secretary.*

